# Leupeptin reduces impulse noise induced hearing loss

**DOI:** 10.1186/1745-6673-6-38

**Published:** 2011-12-29

**Authors:** Haim Gavriel, Abraham Shulman, Alfred Stracher, Haim Sohmer

**Affiliations:** 1 Department of Otolaryngology Head and Neck Surgery Assaf Harofeh Medical Center, Zerifin, Israel; 2 Department Otolaryngology-Head & Neck Surgery, State University of New York Downstate Medical Center, Brooklyn, NY 11203, USA; 3 Department of Pharmacology/Physiology, State University of New York Downstate Medical Center, Brooklyn, NY 11203, USA; 4 Dept. of Physiology; Institute for Medical Research - Israel-Canada, Hebrew University-Hadassah Medical School, Jerusalem, Israel

**Keywords:** protection, noise, apoptosis, threshold shift, calpains, rifle

## Abstract

**Background:**

Exposure to continuous and impulse noise can induce a hearing loss. Leupeptin is an inhibitor of the calpains, a family of calcium-activated proteases which promote cell death. The objective of this study is to assess whether Leupeptin could reduce the hearing loss resulting from rifle impulse noise.

**Methods:**

A polyethelene tube was implanted into middle ear cavities of eight fat sand rats (16 ears). Following determination of auditory nerve brainstem evoked response (ABR) threshold in each ear, the animals were exposed to the noise of 10 M16 rifle shots. Immediately after the exposure, saline was then applied to one (control) ear and non-toxic concentrations of leupeptin determined in the first phase of the study were applied to the other ear, for four consecutive days.

**Results:**

Eight days after the exposure, the threshold shift (ABR) in the control ears was significantly greater (44 dB) than in the leupeptin ears (27 dB).

**Conclusion:**

Leupeptin applied to the middle ear cavity can reduce the hearing loss resulting from exposure to impulse noise.

## Background

Exposure to continuous noise can induce a hearing loss (noise induced hearing loss = NIHL) which can be temporary (TTS) or permanent (PTS), depending on the intensity of the noise and its duration. Studies with drugs designed to alleviate this hearing loss (HL) have shown that the major mechanism involved in inducing this HL is related to the generation in the ear of excessive levels of free radicals which lead to the breakdown of essential molecules and structures in the inner ear [1,2]. The high levels of free radicals are produced as a byproduct of the elevated metabolism which is needed to maintain the electro-chemical gradients required by the cochlear amplifier in order to induce active displacements of the outer hair cells and the basilar membrane in the cochlea during the noise exposure [3]. Therefore drugs which decrease the active displacements such as salicylic acid [4] and furosemide [1], or anti-oxidants for example N acetyl-L-cysteine (NAC) [5,6] and vitamins A, C, E [7], which counteract free radicals, have been shown to be effective in reducing the resultant HL. These drugs have been shown to provide maximal protection from the noise if they are administered just before the continuous noise exposure (salicylic acid and furosemide) or when their injection begins just before and continues after the exposure (NAC, vitamins).

The present study evaluated the NIHL resulting from exposure to impulse noise and the ability of a drug with a different mechanism of action than the aforementioned drugs to alleviate the resulting HL. In contrast to continuous noise, impulse noise is intermittent. In addition to an intense noise level (which may also lead to synthesis of excessive free radicals, as with continuous noise), impulse noise includes a component of rapid rise in the intensity of the sound pressure, which can cause direct mechanical damage (tearing) to inner ear structures [8,9]. An example of impulse noise is that produced by the firing of an M16 rifle, which reaches peak levels of about 165 dB SPL with a rise time of 88 μsec [10].

The drug used in this study in order to try and reduce the HL resulting from exposure to the impulse noise of an M16 rifle was leupeptin. This drug is an inhibitor of the calpains, a family of calcium-activated proteases which promote cell death as a result of the breakdown of membranes, proteins and transcription factors. The roles of the protease calpain and that of leupeptin have been reviewed [11]. Previous studies have led to the suggestion that calpain may also be involved in NIHL. For example, Haupt and Scheibe [12] reported that in guinea pigs exposed to loud broadband noise, the partial pressure of oxygen in the perilymph and the cochlear blood flow were reduced. This may promote calpain up-regulation in early stages of apoptotsis within the organ of Corti. In addition, infusion of leupeptin into scala tympani led to a reduction in the hearing loss (assessed with the auditory evoked response) resulting from a 14 day exposure of chinchillas to a 100 dB SPL octave band noise centered at 4.0 kHz [13].

It has been reported that leupeptin applied to organ cultures from the cochlea, from utricular maculae and from the crista of the semicircular canals, was able to reduce the hair cell loss resulting from addition of gentamicin to the organ cultures [14]. Furthermore, when the drug was infused into the inner ear, it led to a reduction in the amount of hair cell loss following exposure to noise [15]. Since cell death (outer hair cells) following noise exposure occurs at a later stage in the development of HL, it was thought that leupeptin may possibly rescue the sensory epithelium in the cochlea.

Accordingly, experimental animals were exposed to M16 rifle shots and then a non-toxic concentration of leupeptin was applied to one middle ear and saline to the opposite ear. Several days later, the threshold shift in the leupeptin ear was smaller than that in the saline (control) ear.

## Materials and methods

All experimental procedures were authorized by the Hebrew University- Hadassah Medical School Animal Care and Use Committee.

The present study consisted of two phases.

### General description for both phases

The first phase was a functional assessment of hearing following application of various concentrations of leupeptin to the middle ear cavity in order to assess possible ototoxicity and to determine the non-toxic concentration of the drug to be applied to the ear in the second phase of the study. The second phase was to investigate the potential neuroprotective effect of non-toxic concentrations of leupeptin introduced to the middle ear cavity after exposure to traumatic impulse noise.

The overall study was conducted on a total of 20 adult fat sand rats (Psammomys obesus) with a mean body weight of 219 g (200 g to 260 g). The fat sand rat is a rodent species found in the deserts of the Middle East and northern Africa. The frequency range of highest auditory sensitivity in this species is between 0.5 to 5 kHz [16], lower than that in other rodents, and similar to that of humans. It was chosen for the present study because of its unique middle and inner ear anatomy consisting of a large bulla cavity, a thin otic capsule, and an inner ear that clearly projects into the middle ear cavity [17]. This anatomy allows delicate middle and inner ear procedures. In addition, our laboratory has extensive experience in induction and recording of short-latency auditory evoked potentials in this rodent, which were used in this study for functional evaluation of the auditory system.

Solutions of leupeptin and additional relevant drugs were administered into the middle ear of the animals through an implanted polyethylene tube. In order to introduce the tube, the animals were anesthetized with intraperitoneal injection of 25 mg/kg pentobarbital and additional doses were given intraperitoneally as needed. While the animals were under anesthesia, rectal temperature was monitored using a thermistor probe (Yellow Spring Instruments, Yellow Springs, OH) and maintained at 37°C ± 0.5°C (using heating pads).

All animals underwent bilateral introduction of the polyethylene tube into the middle ear cavity for repetitive application of a drug solution into the middle ear. The surgical procedure included a small incision behind the pinna of the ear, and exposure of the bone. A small hole in the bone of the cortex was created between the superior and inferior horizontal septa. After visualizing the round window, a 1.5-cm length of polyethylene tube (external diameter 1.27 mm; internal diameter 0.86 mm) was inserted through the small hole in the bone with one end in position opposite the round window, and the other end of the tube was then fixed externally to the bone with glue and to the skin with 3-0 silk suture.

Auditory function was assessed by recording the auditory nerve-brainstem evoked responses (ABR) in response to alternating polarity broadband clicks presented at a rate of 20.6 clicks per second from an intensity of 120 dB peak equivalent (pe) sound pressure level (SPL) down to threshold in 5-dB steps by an insert earphone within the external ear canal of the studied ear. The ABR was elicited and evaluated using standard clinical equipment (Navigator Pro System, Biological Systems Corporation, Mundelein, Illinois, USA), with recording subdermal needle electrodes (Grass Instrument Division, Astro-Med Inc., West Warwick, RI, USA) at the vertex referred to the chin, and a ground electrode in the left hindlimb. The recorded activity was bandpass filtered (300-3,000 Hz) and averaged (N = 128). Threshold was defined as the lowest intensity that elicited repeatable responses in at least three repeated measurements.

### Phase I: Methods: Determination of a non-toxic concentration of leupeptin

This phase was conducted on 6 animals (12 ears) in which a volume of 0.2 cc of different concentrations of leupeptin (15% to 0.01%) dissolved in saline solution was applied to the middle ear cavity through the polyethylene tube. In several animals, a different concentration of leupeptin was applied to each ear (see table 1). Six additional animals served as controls for this phase. The six control animals received 0.2 cc saline solution to the left middle ear, and 0.2 cc of 40 mg/ml gentamicin to the right middle ear, as a known ototoxic control to confirm that the drugs applied to the middle ear could penetrate the inner ear (presumably through the round window) and affect it. All drugs (leupeptin, gentamicin, saline) were applied once every day for five consecutive days. ABR was again recorded in the surviving animals 3 days after the final application, i.e. 8 days after the first administration. Subsequently, a lethal dose of pentobarbital was injected intraperitoneally. A postmortem examination of the middle ear was conducted to visually assess the effect of substances on middle ear tissue and to confirm that the polyethylene tube was still in place.

**Table 1:** Assessment of toxicity of various concentrations of leupeptin and other agents on ABR thresholds before and 3 days after middle ear application for 5 consecutive days.

Agent	Concentration	No Ears	ABR Threshold (dB pe SPL)*		Clinical observation
				
			Baseline	After Application	
Leupeptin	15%	3**	55	120	Ipsilateral head tilt, bloody otorrhea
	
	1%	2	55	115	Bloody otorrhea
	
	0.1%	3**	56.7	60	Bloody otorrhea
	
	0.01%	3**	50	55	

Gentamicin	4%	6	50	111.6	

Saline	0.9%	6	50	56.7	

* No response was calculated as 120 dB

**One animal died after 3^rd ^injection.

### Phase I: Results: Determination of a non-toxic concentration of leupeptin

Leupeptin was applied to the middle ear through the polyethylene tube at several concentrations (beginning with 15%, down to 0.01%) to determine its possible systemic and ototoxic effects. The ABR threshold before application of any concentration of leupeptin (baseline) was between 50 to 60 dB pe SPL (see table 1). After application of leupeptin at a concentration of 15% to the right middle ear of one animal (1% was applied to its left ear), a right head tilt was observed. Bloody otorrhea was detected from the second day of leupeptin application in all ears injected with 15% leupeptin. After 5 consecutive applications, ABR could not be recorded in the 2 ears remaining in this group which received 15% leupeptin (one animal died after the third injection). In 3 ears treated with middle ear application of leupeptin at a concentration of 1%, bloody otorrhea was detected from the 2^nd ^day of leupeptin application. After a period of 5 consecutive days of application, ABR could not be recorded in one ear and a threshold elevation to 110 dB SPL in the second ear was observed. After middle ear application of leupeptin at a concentration of 0.1%, bloody otorrhea was also detected from the 2^nd ^day of leupeptin application. However, after a period of 5 consecutive days of once a day application of 0.1% leupeptin, a non-significant elevation of the ABR thresholds to 60 dB SPL in 2 ears was observed. Finally, after middle ear application of leupeptin at a concentration of 0.01%, no otorrhea was detected, and after a period of 5 consecutive days of application, ABR was not significantly elevated (it reached 60 dB SPL in 2 ears). Therefore this concentration (0.01%) was used in the second phase of this study.

Post mortem examination revealed normal tympanic membrane and normal middle ear anatomy and mucosa in all ears that received leupeptin.

### Ototoxic control-Results

#### Saline solution (control group)

The baseline mean ABR threshold of 50 dB SPL before saline application (baseline) did not change significantly (56.7 dB SPL) after saline application (see table 1).

#### Gentamicin (ototoxic control group)

After gentamicin application, ABR waves could not be recorded in one ear and thresholds were significantly elevated to a mean value of 111.6 (± SD 6.2) dB SPL in the other 5 ears.

Post mortem examination revealed normal tympanic membrane and normal middle ear anatomy and mucosa in all ears that received gentamicin.

### Phase II: Methods: Protective effect of leupeptin

Unsuccessful attempts had been made to elicit a permanent threshold shift (PTS) in animals following their exposure to *simulated *M16 rifle impulse noise obtained from an internet sound effects site, with amplifiers and loud speakers. The peak intensity of these simulated M16 shots either did not reach the desired intensity of 165 dB SPL or the rise time was lower than the 88 μsec of actual M16 shots [18]. Therefore, the noise exposure in the present study was that of real M16 rifle shots during target practice sessions.

The experimental group consisted of 8 animals (16 ears) in which the polyethylene tube was introduced bilaterally into the middle ear. ABR threshold was recorded in each ear immediately after tube insertion, and again, 8 days after exposure to noise.

The animals were exposed to ten M-16 gunshots. The exposure level was about 165 dB SPL. The experimenter was equipped with ear protectors during the exposure. An attempt to measure the intensity of the impulse noise was made using a Bruel &Kjaer, type 2218, precision integrating sound level meter (Naerum, Denmark). It was necessary to extend the range of the sound level meter. A cover for the microphone was fashioned from Mack's earplugs (McKeon Products, Inc., Madison Heights, MI) material, providing a sound attenuation of about 20 dB.

Immediately after exposure, and once a day over the following 3 days (a total of 4 applications to each ear for each animal), all animals received 0.2 cc of 0.01% (in saline) of leupeptin to the right middle ear cavity and 0.2 cc saline solution to the left middle ear, through the polyethylene tubes. The final ABR threshold was assessed 8 days after the impulse noise exposure. The differences (final minus initial thresholds) between the ABR thresholds (in the ears treated with leupeptin and separately in those given saline) after impulse noise exposure were analyzed statistically using a 2-tailed paired t test. A post mortem was then conducted at the end of the experiment, examining the status of the tube and of the middle ear.

All experimental procedures were authorized by the Hebrew University- Hadassah Medical School Animal Care and Use Committee.

### Phase II: Results: Protective effect of leupeptin-

The mean ABR threshold before exposure (baseline) in the 8 ears which were to receive saline following the M16 impulse noise exposure was 51.3 ± 7.8 dB pe SPL. The mean ABR threshold before exposure (baseline) in the ears to be administered leupeptin after the exposure was 51.3 ± 3.3 dB pe SPL (table 2).

**Table 2:** ABR threshold (in dB pe SPL) before and 8 days after M16 rifle impulse noise exposure and the resulting threshold shift with application of either saline or leupeptin to the middle ear for 4 consecutive days.

Animal	Initial Threshold		Final Threshold*		Threshold Shift	
	
	Saline	Leupeptin	Saline	Leupeptin	Saline	Leupeptin
1	45	50	55	50	10	0

2	50	50	105	120	55	70

3	45	50	115	85	70	35

4	45	50	60	45	15	-5

5	50	50	95	75	45	25

6	50	50	120*	80	70	30

7	70	50	120*	80	50	30

8	55	60	90	90	35	30



Mean ± SD	51.3 ± 7.8	51.3 ± 3.3	95.0 ± 23.9	78.1 ± 21.9	43.7 ± 21.1	26.9 ± 21.4

* No response was calculated as 120 dB

One week after exposure to M16 impulse noise with 4 consecutive days of saline solution application into the middle ear cavity through the polyethylene tube beginning immediately after the exposure, the ABR threshold was elevated to a mean value of 95.0 ± 23.9 dB pe SPL; i.e. a mean threshold shift of 44 dB in the saline control ears. However, one week after exposure, following 4 consecutive days of applying 0.01% leupeptin into the middle ear cavity through the polyethylene tube, the ABR threshold was elevated to a mean value of 78.1 ± 21.9 dB SPL; i.e. a mean threshold shift of only 27 dB in the ears in which a 0.01% solution of leupeptin had been administered. The difference in the threshold shift between the two groups (saline and leupeptin) was found to be significant; P = 0.045 (see table 2).

Post mortem examination revealed normal tympanic membrane and normal middle ear anatomy and mucosa in all ears and the absence of fluid in the middle ear.

## Discussion

Noise-induced trauma is one of the most common, preventable causes of sensorineural hearing loss. However, adequate treatment is not yet available and the noise exposure causes apoptosis in the auditory sensory epithelium. The key proteases that actively participate in the programmed cell death are the calpains, and leupeptin has been shown to be able to protect auditory hair cells from acoustic overexposure when infused directly into the scala tympani of animals prior to noise exposure [13]. However, intracochlear infusion is not a treatment modality in humans, since it involves destruction of the inner ear, thus making it not feasible in humans. Also the presently available form of leupeptin cannot be administered systemically. Hence, a more practical approach involving application of the drug to the middle ear was used in the present study. Application of drugs through a small perforation of the tympanic membrane is used as a treatment option, e.g. in sudden sensory hearing loss and Meniere disease [19-21].

In this study leupeptin was applied directly into the middle ear by means of an implanted polyethelene tube, one end of which reached the middle ear cavity opposite the round window, while the other end was externally accessible. From the middle ear cavity, the drug was able to reach the inner ear, presumably by diffusion through the round window, as shown by the result that a solution of the known ototoxic drug gentamicin with a molecular weight similar to that of leupeptin (427 to 478) applied in the same way, caused a profound HL. The form of leupeptin administered in this study could not have been injected systemically, only topically to the middle ear. Even though the drug solution was applied locally, higher concentrations had systemic toxic effects, not only local effects to the ear. Concentrations of leupeptin which were not systemically or locally toxic (0.01%) were effective in protecting the inner ear from the M16 impulse noise; i.e. the ears to which the drug was applied had a significantly smaller mean threshold shift than that in the opposite ear (control) in the same animal to which a saline solution was applied (27 dB compared to 44 dB).

Leupeptin has been studied extensively in animal models to study Calpain inhibition and its tissue protective effect [11]. No evidence of toxicity has been observed in any of these studies at the concentration and mode of administration used (intraperitoneal, intramuscular, oral). It was also not ototoxic when applied for eight weeks to the round window [22]. In the present study and one other [23], some evidence of toxicity was observed at extremely high doses as well as when a mini pump was used to administer the drug directly into the cochlea (presumably causing a high intracochlear concentration of the drug).

In this study, the solutions were applied immediately after the noise exposure and for the following three days (a total of four applications). The efficacy of the drug solutions applied before the exposure was not assessed because in such a case, a conductive HL would have been present during the noise exposure, reducing the effectiveness of the noise exposure. A conductive HL was probably not present at the time of the final threshold determination (three days after the last drug application) since post-mortem examination of the middle ear found it clear of fluid.

Leupeptin, a potent inhibitor of calpains (calcium activated proteases which promote breakdown of proteins, several enzymes and transcription factors, culminating in cell death) applied to the middle ear cavity leads to a significant reduction in the threshold shift caused by exposure to impulse noise.

In future studies, we intend to administer a form of leupeptin which can be given systemically and then to assess its possible systemic toxicity and ototoxicity (overall, the systemic toxicity and ototoxicity would be dependent on the concentration of the drug at each delivery, the time between successive applications, the concentration of the drug in blood and the total number of injections), its efficacy compared to other drugs in protecting from continuous and impulse noise and to determine the optimal time window for administration (either before, or at several time periods after the exposure) in order to obtain a maximal degree of rescue.

## Conclusion

Leupeptin applied to the middle ear cavity can reduce the hearing loss resulting from exposure to impulse noise.

## Competing interests

There is no direct or indirect commercial financial incentive associated with publishing the article; there is no extra-institutional funding; there are no possible conflicts of interest; there are no sources of financial support, corporate involvement, patent holdings, etc for our research/study; and there is no ethical problem. There are no non-financial competing interests (political, personal, religious, ideological, academic, intellectual and commercial).

## Authors' contributions

HG have made substantial contributions to conception and design, to acquisition of data and analysis and interpretation of data; have been involved in drafting the manuscript, and gave final approval of the version to be published.

AS (Shulman) have made substantial contributions to the design and interpretation of data; have been involved in revising the manuscript and gave final approval of the version to be published.

AS (Stracher) have made substantial contributions to conception and design; have been involved in revising the manuscript critically for important intellectual content and gave final approval of the version to be published.

HS have made substantial contributions to conception and design and to analysis and interpretation of data; have been involved in drafting the manuscript or revising it critically for important intellectual content; and gave given final approval of the version to be published.
